# Role of 5-Hydroxytryptamine 1A Receptors in 6-Hydroxydopmaine-induced Catalepsy-like Immobilization in Rats: a Therapeutic Approach for Treating Catalepsy of Parkinson’s Disease 

**Published:** 2012

**Authors:** Siamak eyhani-rad, Alireza Mohajjel Nayebi, Javad Mahmoudi, Morteza Samini, Vahab Babapour

**Affiliations:** a *Science and Research Branch, Islamic Azad University, Tehran, Iran.*; b *Department of Pharmacology and Toxicology, Faculty of Pharmacy, Tabriz University of Medical Sciences. *; c *Drug Applied Research Center, Tabriz University of Medical Sciences. *d*Neurosciences *; d *Research Center (NSRC), Tabriz University of medical Sciences, Tabriz Iran. *

**Keywords:** 5-HT1A, 6-Hydroxydopamine, 8-OHDPAT, NAN-190, Catalepsy

## Abstract

We have shown that buspirone, a partial agonist of 5-hydroxytryptamine 1A (5-HT1A) receptors, improves motor dysfunctions induced by 6-hydroxydopamine (6-OHDA) and haloperidol in rats. The present work extends these findings by investigating the role of 5-HT1A receptors on catalepsy-like immobilization in rats, a model of Parkinson’s disease. Catalepsy was induced by unilateral infusion of 6-OH-dopamine (8 μg/2μL/rat) into the central region of the substantia nigra, compact part (SNc) and assayed by bar-test method 5, 60, 120 and 180 min after the drugs administration. The involvement of 5-HT1A receptors in 6-OHDA-induced catalepsy was studied through intraperitoneal (0.25, 0.5 and 1mg/Kg IP) and intrasubstantia nigra, compact part (10 μg/rat, intra-SNc) injection of 8-hydroxy-2-[di-n-propylamino] tetralin (8-OHDPAT) as well as administration of 1-(2-methoxyphenyl)-4-[4-(2-pthalimmido) butyl] piperazine hydrobromide (0.1, 0.5 and 1 mg/Kg, NAN-190, IP). NAN-190 (1 mg/Kg, IP) and 8-OHDPAT (1 mg/Kg, IP and 10 μg/rat, intra-SNc) increased and decreased 6-OHDA-induced catalepsy respectively. In normal (non 6-OHDA-lesioned) rats, NAN-190 (1 mg/Kg, IP) increased the elapsed time in bar-test while 8-OHDPAT did not produce any significant effect. The anticataleptic effect of 8-OHDPAT (1 mg/Kg, IP) was reversed markedly by co-injection with NAN-190 (1 mg/Kg, IP). These findings suggest that 5-HT1A receptors are involved in 6-OHDA-induced catalepsy-like immobilization.

## Introduction

Parkinson’s disease (PD) is a neurodegenerative disease mainly caused by degeneration of dopaminergic neurons from the substantia nigra pars compacta (SNc). This causes the loss of dopamine (DA) release in the corpus striatum, the brain area that receives the projections from the nigral dopaminergic neurons (1). The resulting deficiency of DA in the striatum leads to hypokinetic motor function characterized by rigidity, tremor, bradykinesia and postural instability (1, 2). However, other neurotransmitter systems also show signs of degeneration or hyperinnervation, among which is the serotonergic system (2, 3). Studies on the role of other neurotransmitters have been performed with the intent of developing adjunct antiparkinsonian treatments that do not act on DA pathways.

Serotonergic (5-HT) pathway can have regulatory effects on DA-mediated motor function that may be useful in treating the symptoms of PD (4). It has been shown that 5-HT1A receptors are located on dorsal raphe neurons with efferents to the striatum, and are also localized on cortical neurons sending glutamatergic projections to the basal ganglia (5). 5-HT1A autoreceptors exist on the serotonergic cell soma and dendrites in the raphe nuclei where they can reduce 5-HT synthesis and serotonergic transmission in terminal field (6). Studies have shown that 5-HT1A receptor stimulation represents antiparkinsonian effects in 6-hydroxydopamine (6-OHDA) lesioned rats (7-9). Recently, we have reported that buspirone as a partial agonist of 5-HT1A receptors, improves catalepsy induced by 6-hydroxydopamine (10) and haloperidol (11) as animal models of Parkinson’s disease. This effect is most likely caused by the increase in 5-HT1A receptor activation, resulting in an inhibition of serotonin release (6). Stimulation of 5-HT1A receptor is associated with an increase in dopamine turnover (12), dopaminergic cell firing (13) and dopamine release (14) suggesting that 5-HT1A agonists might have potential therapeutic value in the treatment of Parkinson’s disease. We have hypothesized that 5-HT1A receptor agonist may have potential therapeutic effect in alleviating the motor symptoms of Parkinson’s disease and the augmentation of these agents to antiparkinson and neuroleptic drugs may increase the efficiency of antiparkinson and neuroleptic drugs (15). This study is in extension of our recent works and was designed to further substantiate role of 5-HT1A receptors in catalepsy induced by 6-OHDA.

## Experimental

*Chemicals*

All chemicals were obtained from Sigma Chemical Company (USA). Solutions were prepared fresh on the days of experimentation. 8-hydroxy-2-[di-n-propylamino] tetralin (8-OH-DPAT) and 1-(2-methoxyphenyl)- 4-[4-(2-pthalimmido) butyl] piperazine hydrobromide (NAN-190) were dissolved in physiological saline (0.9% NaCl) and 6-hydroxydopamine (6-OHDA) was dissolved in 0.9% saline containing 0.2% (w/v) ascorbic acid. The drugs were injected into the central region of the substantia nigra pars compacta (SNc) in a total volume of 2 μL/rat with a constant injection rate of 0.2 μL/min and by IP route in a volume of 0.1 mL.

*Animals*

The experiments were carried out on male Wistar rats weighting 180-200 g. Animals were housed in standard polypropylene cages, four per cage, under a 12:12 h light/dark schedule at an ambient temperature of 25 ± 2°C and had access to food and water ad libitum. Animals were accustomed to the testing conditions for 2 days before the behavioral experiment was conducted. All the procedures were carried out under the ethical guidelines of the Tabriz University of Medical Sciences, according to the guide for the care and use of laboratory animals (National Institutes of Health Publication No 85-23, revised 1985).

*Surgical procedures*

Animals were anaesthetized by intraperitoneal (IP) injection of sodium thiopental (40 mg/Kg), and additional anesthetics (4 mg/Kg, IP) were given when necessary. After being deeply anaesthetized (loss of corneal and toe pad reflexes), rats were mounted in a stoelting (USA) stereotaxic frame in the flat skull position. The scalp was shaved, swabbed with iodine and a central incision made to expose the skull. Cannula (23 gauge stainless steel) was implanted to serve as the guide for subsequent insertion of injection tube into the substantia nigra compact part (SNc). The coordinates for these sites were based on rat brain atlas (16) : anteroposterior (AP) -5.0 mm from the bregma; mediolateral (ML) -2.1 mm from midline and dorsoventral (DV) -7.7 from the skull. The guide cannula was then secured to the cranium with dental cement. Sham-operated animals were submitted to the same procedure but 2 μL vehicle (0.9% saline containing 0.2% (w/v) ascorbic acid was infused into the SNc. *Histology*

All rats with guide cannulas were sacrificed at the end of the experiments. The brain dissects were prepared for all animals to confirm the exact implantation of guide cannula in SNc. The brains were fixed in 10% formalin for a week with the injecting tube *in-situ*. The location of the tip of the injecting tube was verified in serial sections. Only the results from catalepsy tests of animals in which the tip of the injecting tube was within the SNc area were used for statistical analysis.

*Lesion of dopaminergic neurons*

To induce dopaminergic lesion, 6-OHDA was injected unilaterally into the SNc through the implanted guide cannula at dose of 8 μg/2μL/rat with a rate of 0.2 μL/min. 6-OHDA. Lesioned rats were submitted to subsequent the experiments, 5 days after the surgery as a recovery period.

*Catalepsy test*

Catalepsy was measured by means of a standard bar-test, as the time the rats maintained an imposed position with both front limbs extended and restring on a 9-cm high round wood bar (0.9 cm in diameter). The end point of catalepsy was considered to occur when both front paws were removed from the bar or if the animal moved its head in an exploratory manner. The cut-off time of the test was 720 sec. This test was carried out 5, 60, 120 and 180 min after the drugs administration. All observations were made between 9 AM and 4 PM by an observer who was blind to the treatments.

*Statistical analysis*

Descriptive statistics and comparisons of differences between each data set were calculated using SigmaStat software. The data was expressed as mean ± SEM, and analyzed by one-way ANOVA in each experiment. Statistical significance was accepted at the level of p < 0.05. In the case of significant variation (p < 0.05), the values were compared by Tukey test.

## Results

*6-OHDA-induced catalepsy*

**Figure 1:**
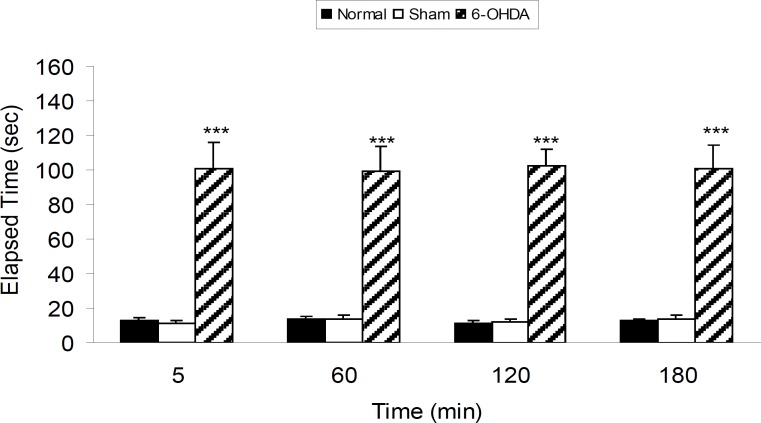
The results of bar-test in normal, sham-operated and 6-OHDA (8 μg/2μL/rat) lesioned rats in different time points after the treatment. Each bar represents the mean ± SEM of elapsed time (s), n = 8 rats for each group: *** p < 0.001 when compared with normal and sham-operated groups

Three groups of rats were subjected as normal, sham operated (receiving 2 μL vehicle of 6-OHDA) and 6-OHDA (8 μg/2 μL/rat) injected. Drugs and vehicle were injected into the SNc through the implanted guide cannula. According to the obtained results, 6-OHDA induced significant (p < 0.001) catalepsy in comparison with both normal and sham-operated rats (Figure 1).

*Effect of 8-OHDPAT (IP. and intra-SNc) on 6-OHDA-induced catalepsy*

**Figure 2:**
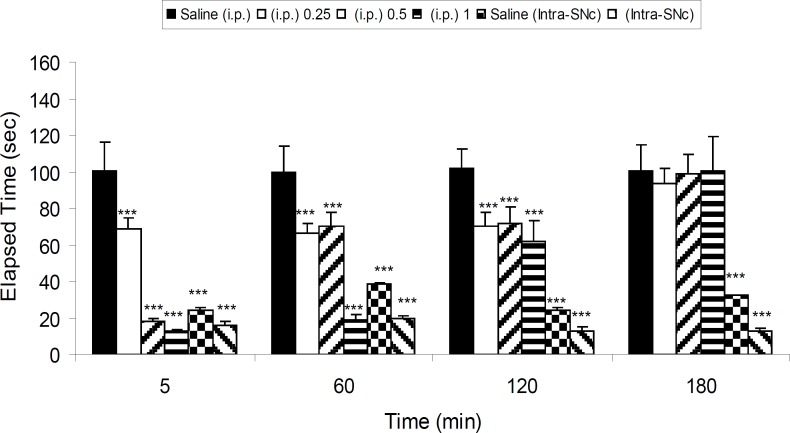
The effect of IP (0.25, 0.5 and 1 mg/Kg) and intra-SNc (10 μg/rat, intra-SNc) injections of 8-OHDPAT on bar test in 6-OHDA-lesioned rats in different time points after the treatment. Each bar represents the mean ± SEM of elapsed time (s), n = 8 rats for each group; *** p < 0.001 when compared with saline-treated groups (Parkinsonian rat).

Five groups of 6-OHDA-lesioned rats received saline (IP), three different IP doses of 8-OHDPAT (0.25, 0.5 and 1 mg/Kg, IP) and one intra-SNc dose (10 μg/rat, intra-SNc) of 8-OHDPAT, respectively. The results showed that 8-OHDPAT both in IP and intra-SNc routes was able to attenuate the severity of 6-OHDA-induced catalepsy (p < 0.001; Figure 2).

*Effect of NAN-190 on 6-OHDA-induced catalepsy*

**Figure 3:**
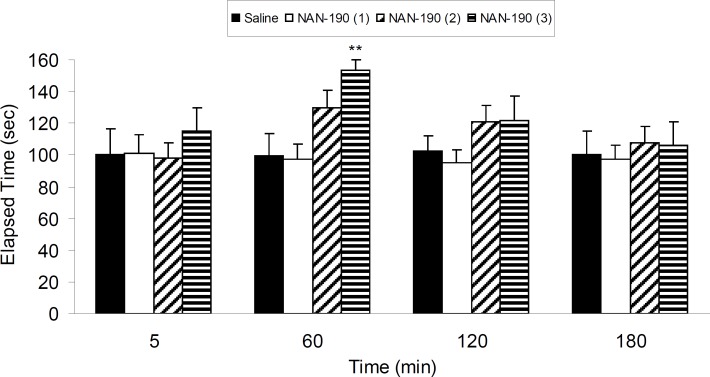
The results of bar-test in 6-OHDA-lesioned rats treated with three different IP doses of NAN-190 (0.1, 0.5 and 1 mg/Kg) in different time points after the treatment. Each bar represents the mean ± SEM of elapsed time (s), n = 8 rats for each group;** p < 0.01 when compared with saline-treated group

Four groups of 6-OHDA-lesioned rats were treated with three different IP doses of NAN-190 (0.1, 0.5 and 1 mg/Kg) and vehicle. Results show that NAN-190 (1 mg/Kg, IP) increased (p < 0.01) 6-OHDA-induced catalepsy compared with vehicle-treated group (Figure 3).

*Effect of NAN-190 co-treatment with 8-OH-DPAT on 6-OHDA-induced catalepsy*

**Figure 4:**
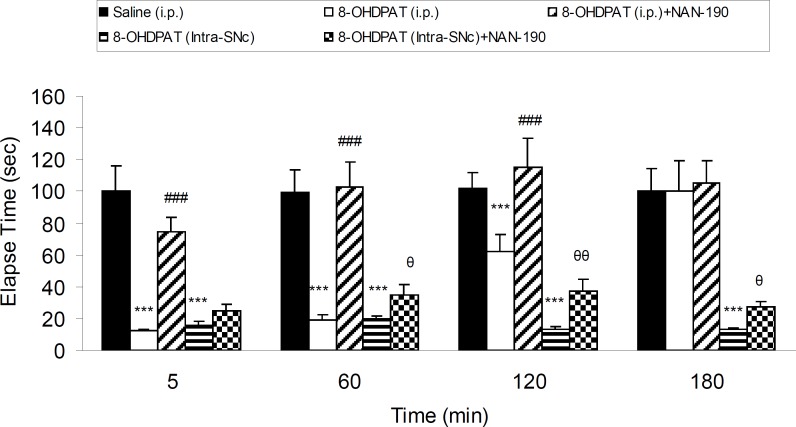
The effect of NAN-190 (0.5 mg/Kg, IP) co-treatment with 8-OHDPAT (1 mg/Kg, IP and 10 μg/rat, intra-SNc) and NAN-190 (10 μg/rat, intra-SNc ) on bar-test in 6-OHDA-lesioned rats in different time points after the treatment. Each bar represents the mean ± SEM of elapsed time (s), n = 8 rats for each group; *** p < 0.001 when compared with saline-treated group. ### p < 0.001 when compared with 8-OHDPAT (1 mg/Kg IP) treated group. θθ: p < 0.01 , θ: p < 0.05 when compared with 8-OHDPAT (Intra-SNc) treated group

Five groups of 6-OHDA-lesioned rats received saline (IP), 8-OHDPAT (1 mg/Kg, IP), 8-OHDPAT (1 mg/Kg, IP) and NAN-190 (0.5 mg/Kg, IP), 8-OHDPAT (10 μg/rat, intra-SNc) and 8-OHDPAT (10 μg/rat, intra-SNc) with NAN-190 (10 μg/rat, intra-SNc) respectively. Results showed that anti-cataleptic effect of 8-OHDPAT both in IP (1 mg/Kg) and intra-SNc (10 μg/rat) was abolished significantly (p < 0.05, p < 0.01, p < 0.001) by NAN-190 (Figure 4).

## Discussion

In this study, the effect of 8-OHDPAT, 5-HT1A-receptor agonist was investigated in 6-OHDA-lesioned rats. 6-OHDA is a neurotoxin which is used commonly to create experimental models of PD. Our results showed that 6-OHDA was able to induce catalepsy when injected unilaterally into the substantia nigra compact part (SNc). Catalepsy was assessed by the bar-test which is a standard test frequently used for the evaluating of catalepsy-like immobilization induced by 6-OHDA and neuroleptic drugs in rodents (17). We observed that 8-OHDPAT, an agonist of 5-HT1A-receptors, improved catalepsy in 6-OHDA-lesioned rats. This is in accordance with previous studies reporting a possible role for 5-HT1A agonists in decreasing the motor abnormalities associated with PD (7-9, 15).

Behavioral and neurochemical studies have revealed that serotonergic system is able to modulate the brain dopaminergic system (18). It has been shown that the 5-HT1A-receptor is present on dorsal raphe neurons with efferents to the striatum, and on cortical neurons sending glutamatergic projections to the basal ganglia (2, 5, 19, 20). Stimulation of 5-HT1A-receptors in these regions leads to dopamine release (15) by the inhibition of adenyl cyclase and the opening of potassium channels (21). These findings indicate that the modulation of 5-HT transmission by 5-HT1A-receptor agonist may be a possible therapeutic approach in Parkinson’s disease. 

8-OHDPAT is a 5-HT1A-receptor agonist which may interact slightly with 5-HT7 and/or α2 adrenergic receptors (22). In order to rule out the involvement possibility of these receptors in the observed effect, we investigated the effect of 8-OHDPAT and NAN-190 (5-HT1A- receptor antagonist) co-administration in catalepsy elapsed time in 6-OHDA-lesioned rats. According to our results, 8-OHDPAT improved 6-OHDA-induced catalepsy both in intraperitoneal and intra-nigral routes. The anti-cataleptic effect of 8-OHDPAT in both routes of administration was abolished by NAN- 190. These results confirm the involvement of 5-HT1A receptors in the observed effect of 8-OHDPAT. We suggest that 8-OHDPAT ameliorates catalepsy in 6-OHDA-lesioned rats by stimulating 5-HT1A receptors of SNc. Therefore, 5-HT1A receptor agonists could potentially be used as an adjuvant therapy together with routinely used antiparkinsonian drugs. In this regard, several studies have revealed that 5-HT1A receptor agonists could be used prophylactically to reduce L-DOPA-induced dyskinesia by stimulating 5-HT1A inhibitory autoreceptors within the raphe nuclei, which may normalize the amount of DA delivered to the striatum (23). 5-HT1A agonists, such as buspirone, have prominent anxiolytic effect and are used clinically for managing some psychiatric disorders (24, 25). Anxiety is one of the most common psychiatric problems experienced by patients with Parkinson’s disease (26). Adjuvant therapy with 5-HT1A agonists may offer an appealing strategy for improving the efficacy of antiparkinson drugs while taking advantage of these anxiolytic effects. 

Most of the typical antipsychotics which are antagonists of D2 receptors, are known to induce extrapyramidal side-effects in patient suffering from psychiatric illness (27). These effects can be modeled in rodents by measuring catalepsy. It has been reported that selective serotonin reuptake inhibitors attenuate the antipsychotic-induced catalepsy in mice (28). In this study, we showed that 8-OHDPAT alleviated 6-OHDA-induced catalepsy by affecting nigral 5-HT1A receptors. This confirms the results of our previous study reporting that buspirone improves haloperidol (11) and 6-OHDA-induced (10) catalepsy through the activation of 5-HT1A receptors. 

In conclusion, we suggest that 8-OHDPAT decreases catalepsy induced by 6-OHDA. This effect is exerted through the activation of nigral 5-HT1A receptors. Therefore, adjuvant therapy with 5-HT1A agonists might improve the efficiency of antiparkinson drugs. Further clinical investigations are needed to test whether 5-HT1A agonists are useful in increasing the efficacy or decreasing the motor side effects of antiparkinson and typical antipsychotic drugs. 

## References

[1] Mink JW (1996). The basal ganglia: focused selection and inhibition of competing motor programs. Prog. Neurobiol.

[2] Scholtissen B, Verhey FRJ, Steinbusch HWM, Leentjens AFG (2006). Serotonergic mechanisms in Parkinson’s disease: opposing results from preclinical and clinical data. J. Neural Transm.

[3] Brown P, Gerfen CR (2006). Plasticity within striatal direct pathway neurons after neonatal dopamine depletion is mediated through a novel functional coupling of serotonin 5-HT2 receptors to the ERK 1/2 map kinase pathway. J. Com. Neurol.

[4] Vincenzo DM, Massimo P, Ennio E, Giuseppe C, Arcangelo B, Giuseppe DG (2008). Serotonin modulation of the basal ganglia circuitry: therapeutic implication for Parkinson’s disease and other motor disorders. Prog. Brain Res.

[5] Knobelman DA, Kung HF, Lucki I (2000). Regulation of extracellular concentrations of 5-hydroxytryptamine (5-HT) in mouse striatum by 5-HT1A and 5-HT1B receptors. J. Pharmacol. Exp. Ther.

[6] Riad M, Garcia S, Watkins KC, Jodoin N, Doucet E, Langlois X, el Mestinkawy S, Hamon M, dsescarries L (2000). Somatodendritic localization of 5-HT1A and preterminal axonal localization of 5-HT1B serotonin receptors in adult rat brain. J. Com. Neurol.

[7] Bezard E, Gerlach I, Moratalla R, Gross CE, Jork R (2006). 5-HT1A receptor agonist-mediated protection from MPTP toxicity in mouse and macaque models of Parkinson’s disease. Neurobiol. Dis.

[8] Dupre KB, Eskow KL, Negron G, Bishop C (2007). The differential effects of 5-HT1A receptor stimulation on dopamine receptor-mediated abnormal involuntary movements and rotations in the primed hemiparkinsonian rat. Brain Res.

[9] Gerber R, Alter CA, Leibman JM (1988). Rotational behavior induced by 8-hydroxy-DPAT, a putative 5-HT1A agonist, in 6-hydroxydopamine-lesioned rats. Psychopharmacology (Berl).

[10] Nayebi AM, Reyhani RS, Saberian M, Azimzadeh S, Samini M (2010). Buspirone improves 6-hydroxydopamine-induced catalepsy through stimulation of nigral 5-HT1A receptors in rat. Pharmacol. Rep.

[11] Nayebi AM, Sheidaei H (2010). Buspirone improves haloperidol-induced Parkinson’s disease in mice through 5-HT receptors. DARU.

[12] Hamon M, Fattaceini C M, Adrien J, Gallissot MC, Martin P, Gozlan H J (1988). Alterations of central serotonin and dopamine turnover in rats treated with ipsapirone and other 5 hydroxytryptamine1A agonists with potential anxiolytic properties. Pharmacol. Exp. Ther.

[13] Arborelius L, Chergui K, Murase S, Nomikos G G, Hook BB, Chouvet G, Hacksell U (1993). The 5-HT1A receptor selective ligands, (R)-8-OH-DPAT and (S)- UH-301, differentially affect the activity of midbrain dopamine neurons. Naunyn Schmiedebergs Arch. Pharmacol.

[14] Ichikawa J, Meltzer HY (1999). R(+)-8-OH-DPAT, a serotonin 1A receptor agonist, potentiated S(–)- sulpiride-induced dopamine release in rat medial prefrontal cortex and nucleus accumbens but not striatum. J. Pharmacol. Exp. Ther.

[15] Nayebi AM (2010). Hypothesis: A promising effect of 5-HT1A receptor agonists in alleviating motor symptoms of Parkinson’s disease. Afr. J. Pharm. Pharmacol.

[16] Paxinos G, Watson C (2007). The Rat Brain in Stereotaxic Coordinates.

[17] Andreas S (2004). Classic toxin-induced animal models of Parkinson’s disease: 6-OHDA and MPTP. Cell Tissue Res.

[18] Alex KD, Pehek EA (2007). Pharmacologic mechanisms of seretonergic regulation of dopamine neurotransmission. Pharmacol. Ther.

[19] Burnet PW, Eastwood SL, Lacey K, Harrison PJ (1995). The distribution of 5-HT1A and 5-HT2B receptor mRNA in human brain. Brain Res.

[20] Wright D E, Seroogy K B, Lundgren KH, Davis BM, Jennes L (1995). Comparative localization of serotonin1A, 1C and 2 receptor subtype mRNA in rat brain. J. Comp. Neurol.

[21] Harrington MA, Oksenberg D, Peroutka SJ (1988). 5-Hydroxytryptamine 1A-receptors are linked to a G-- adenylate cyclase complex in rat hippocampus. Eur. J. Pharmacol.

[22] Hedlund PB, Kelly L, Mazur C, Lovenberg T, Sutcliffe JG, Bonaventure P (2004). 8-OH-DPAT acts on both 5-HT1A and 5-HT7 receptors to induce hypothermia in rodents. Eur. J. Pharmacol.

[23] Susan HF, Rosalind C, Jonathan M, Brotchie (2008). Parkinson’s disease opportunities for novel therapeutics to reduce the problems of levodopa therapy. Prog. Brain Res.

[24] Filip M, Bader M (2009). Overview on 5-HT receptors and their role in physiology and pathology of the central nervous system. Pharmacol. Rep.

[25] Tunnicliff G (1991). Molecular basis of buspirone’s anxiolytic action. Pharmacol. Toxicol.

[26] Forjaz MJ, Rodriguez BC, Martinez MP (2008). On behalf of the Longitudinal Parkinson’s Disease Patient Study (Estudio Longitudinal de Pacientes con Enfermedad de Parkinson-ELEP) Group: Rasch analysis of the hospital anxiety and depression scale in Parkinson’s disease. Mov. Disord.

[27] Imaki J, Mae Y, Shimizu S, Ohno Y (2009). Therapeutic potential of alpha2 adrenoceptor antagonism for antipsychotic-induced extrapyramidal motor disorders. Neurosci. Lett.

[28] Pires JGP, Bonikoski V, Futuro NHA (2005). Acute effects of selective serotonin reuptake inhibitors on neuroleptic-induced catalepsy in mice. Braz. J. Med. Biol. Res.

